# Painful ulcerations associated with COVID-19 in an adolescent patient: a case report

**DOI:** 10.3389/fdmed.2024.1412439

**Published:** 2024-08-02

**Authors:** David O. Danesh, Kyulim Lee, Rebecca G. Wallihan, Janice A. Townsend, Ira Mulo, Ashok Kumar

**Affiliations:** ^1^ Division of Pediatric Dentistry, College of Dentistry, The Ohio State University, Columbus, OH, United States; ^2^ Division of Dentistry, Nationwide Children’s Hospital, Columbus, OH, United States; ^3^ Department of Pediatrics, College of Medicine, The Ohio State University, Columbus, OH, United States

**Keywords:** COVID-19, oral pathology, pediatric dentistry, diagnostic challenge, oral ulcers

## Abstract

Oral lesions associated with SARS-CoV-2 (COVID-19) include aphthous-like ulcers, herpetiform eruption of vesicles and erosions and other findings. Reactive infectious mucocutaneous eruption (RIME) has recently been used to describe non-*Mycoplasma pneumoniae* pathogens that can lead to rash and mucositis. RIME secondary to SARS-CoV-2 infection is consistent with reports in the literature. The patient in this case report is significant in that it involves only the oral mucosa, although there are cases reported where mucosal involvement is limited to one site. The degree of mucosal involvement in our case report was in the presence of an acute COVID-19 infection without ocular or genital involvement. Oral lesions associated with COVID-19 infection vary in presentation. This paper adds to the understanding of systemic manifestations of COVID-19 infection and provides a reference of clinical findings, management, and interdisciplinary collaboration for caring for this patient.

## 1 Introduction

Oral lesions associated with SARS-CoV-2 (COVID-19) vary in prevalence of presentation and association with systemic manifestations (1–6). A case series reports characteristics of oral lesions in 10 patients with COVID-19 that is consistent with reports of other patients published systematic reviews (3, 4, 6). One systematic review and practical guideline published in 2023 reports the oral manifestations of COVID-19, reviewing 24 studies involving over 2,000 pediatric patients with COVID-19 (5).

The literature reports varying prevalence of oral lesions associated with COVID-19 infections and Current research does not definitively conclude that oral lesions are directly related to COVID-19 (4, 7). Some examples of oral lesions associated with COVID-19 include aphthous-like ulcers, herpetiform eruption of vesicles and erosions, *Candida*-associated lesions, recurrent herpesvirus infection, blisters, dark pigmentations, erythema multiforme-like lesions, white plaques, yellowish ulcers, inflammatory and purulent areas on pharyngeal wall, sialadenitis, overgrowth of filiform papillae, tongue depapillation, necrotizing gingivitis, salivary gland infections, angular cheilitis, hyposalivation, xerostomia, burning mouth sensation, facial pain, and oral submucous fibrosis (1–6).

Reactive infectious mucocutaneous eruption (RIME) has recently been used to describe non-*Mycoplasma pneumoniae* pathogens that can lead to rash and mucositis (8). RIME typically presents with mucositis in at least two or more oral, ocular, and/or genital mucosal tissues, with or without cutaneous involvement, and is associated with a prodrome that includes cough (8–11). Pathogens associated with RIME include *Chlamydophila pneumoniae*, metapneumovirus, parainfluenza virus 2, rhinovirus, influenza B virus, and enterovirus (8, 12, 13). RIME may present similar to, but is distinct from, *M. pneumoniae*–induced rash and mucositis (MIRM), erythema multiforme (EM), and Stevens-Johnson syndrome (SJS)/toxic epidermolytic necrosis (TEN) (8, 12, 13). The mechanism of RIME may involve polyclonal B-cell proliferation antibody production, which leads to immune complex deposition, complement activation, and molecular mimicry resulting in skin damage (12, 14).

## 2 Patient information

A 15-year-old-male presented with symptoms of fever, cough, and congestion, and was diagnosed with COVID-19 and acute bronchitis three days after initial symptoms. The diagnosis of COVID-19 was made via reverse transcription polymerase chain reaction (RT-PCR). The patient was otherwise healthy with a non-contributory medical history. Surgical, family, and social histories were unremarkable with no current medications. The patient was not vaccinated against SARS-CoV-2. There was a reported allergy to bee venom and no known drug allergies. The patient was undergoing comprehensive orthodontic treatment. The patient was prescribed prednisone (20 mg tablet, four times daily for 7 days) and azithromycin (500 mg once for 1 day, then 250 mg once daily for 4 days) by a local emergency department for management of COVID-19.

## 3 Case description

Eight days after diagnosis, the patient reported painful mouth sores that initially presented on the soft palate. Lesions progressed to generalized ulcerations to the upper lip and lower lip. At 10 days post-diagnosis, the patient returned to their primary care physician's office and was prescribed nystatin tablets and Magic Mouthwash.

At 11 days post-diagnosis, the patient returned to a local emergency department due to worsening mouth pain and was prescribed oral acyclovir (400 mg four times daily for 7 days) and a compounded “Magic Mouthwash” (active ingredients are 15 ml of a 1:1:1:1 mixture of 100,000 U/ml Nystatin, diphenhydramine 12.5 mg/ml, alum-magnesium hydroxide, and 2% viscous lidocaine every 6 h).

**Figure 1:**
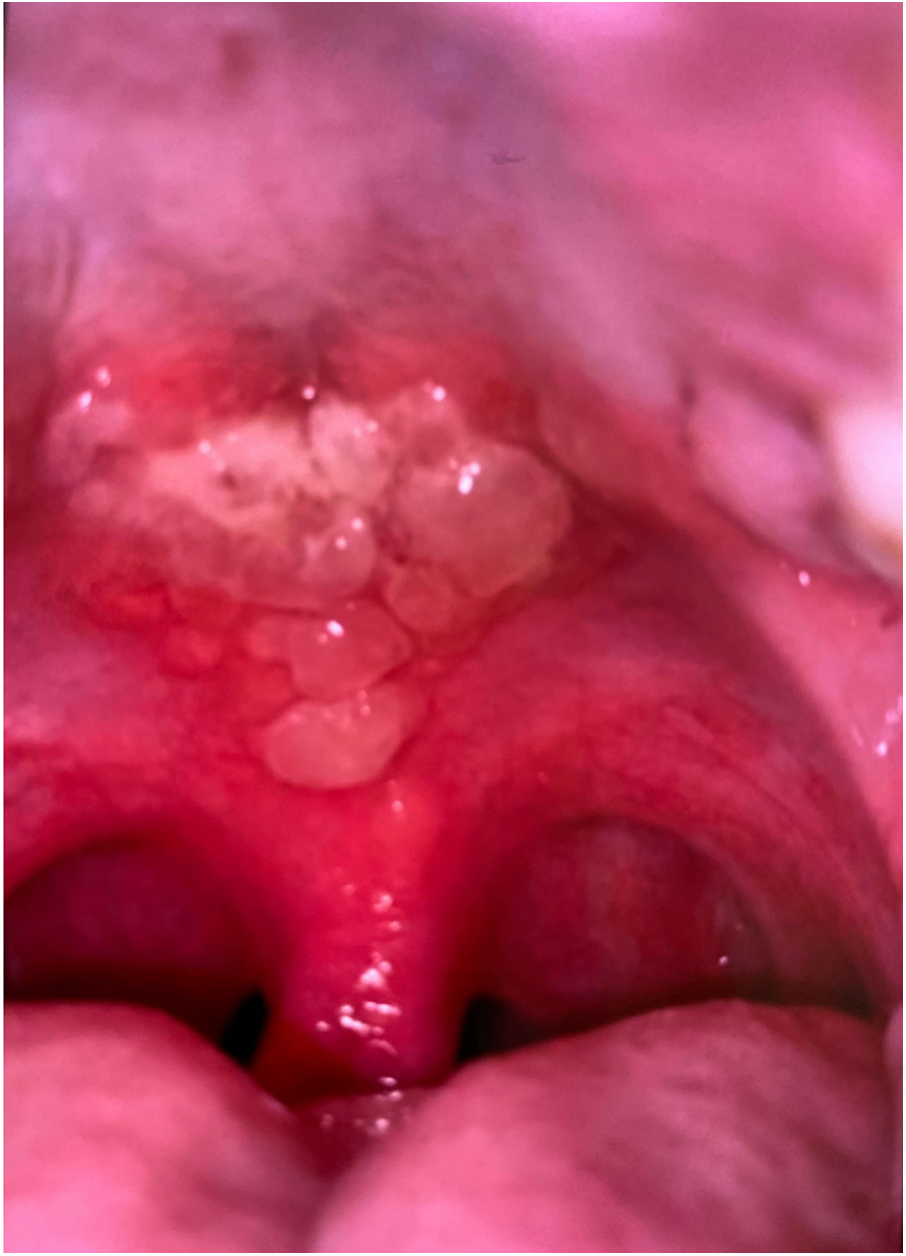
Intraoral photograph, initial presentation for admission, 13 days post-diagnosis, demonstrating mucosal lesions on hard and soft palate.

**Figure 2:**
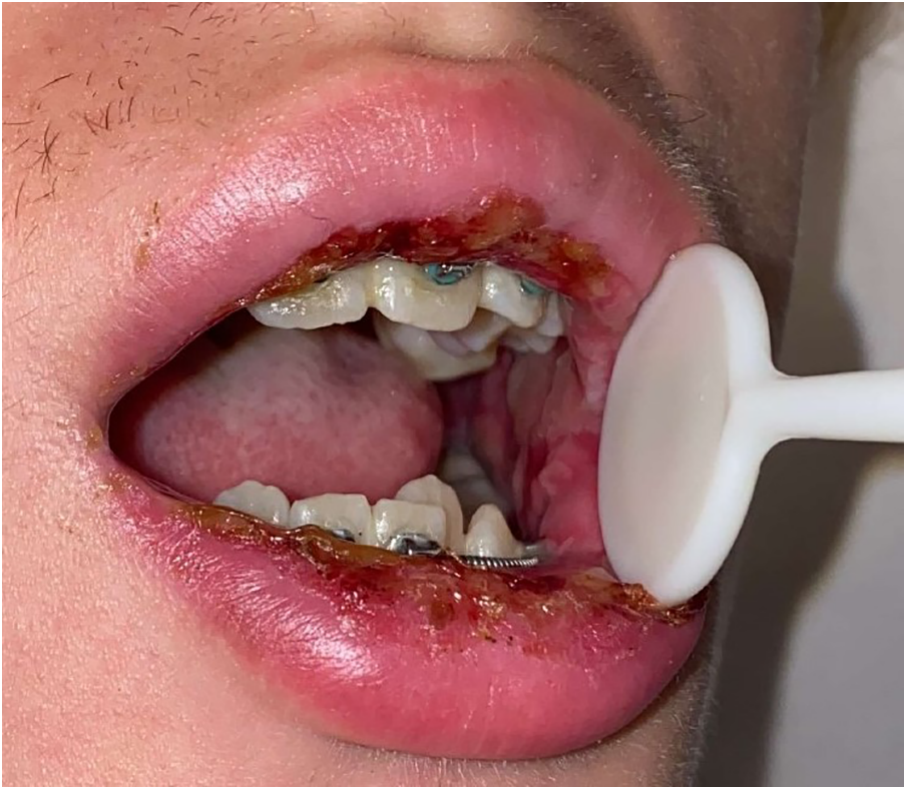
Photograph during admission, 14 days post-diagnosis, frontal view showing extent of mucosal lesions from intraoral to extraoral.

At 13 days post-diagnosis, the patient returned to another emergency department due to severe oral pain (eight out of 10 using the visual analogue pain rating scale), limited opening, and inability to tolerate oral intake. The patient had eight kilograms of weight loss 13 days since the initial COVID-19 diagnosis. Extraoral examination revealed erosive, yellowish ulcerations with crusted surfaces, and sloughing of mucosa on the upper and lower lip vermilion border. Patient had limited opening due to pain from sores. Intraoral examination revealed generalized, wide and shallow, erythematous ulcerations with overlying sloughing mucosa with a moderately coated dorsum of tongue (Figure 1) and numerous, localized, raised fluid-filled vesicular lesions on the posterior soft palate (Figure 2). Slight ulcerations were noted in the floor of the mouth. The patient denied eye pain or genital pain. No additional mucosal membrane involvement was noted. Further testing with a respiratory infection PCR array at 13 days post-diagnosis did not detect rhinovirus, enterovirus, influenza virus, parainfluenza, respiratory syncytial virus, *Bordetella pertussis*, *Chlamydophila pneumoniae*, or *M. pneumoniae*.

**Figure 3:**
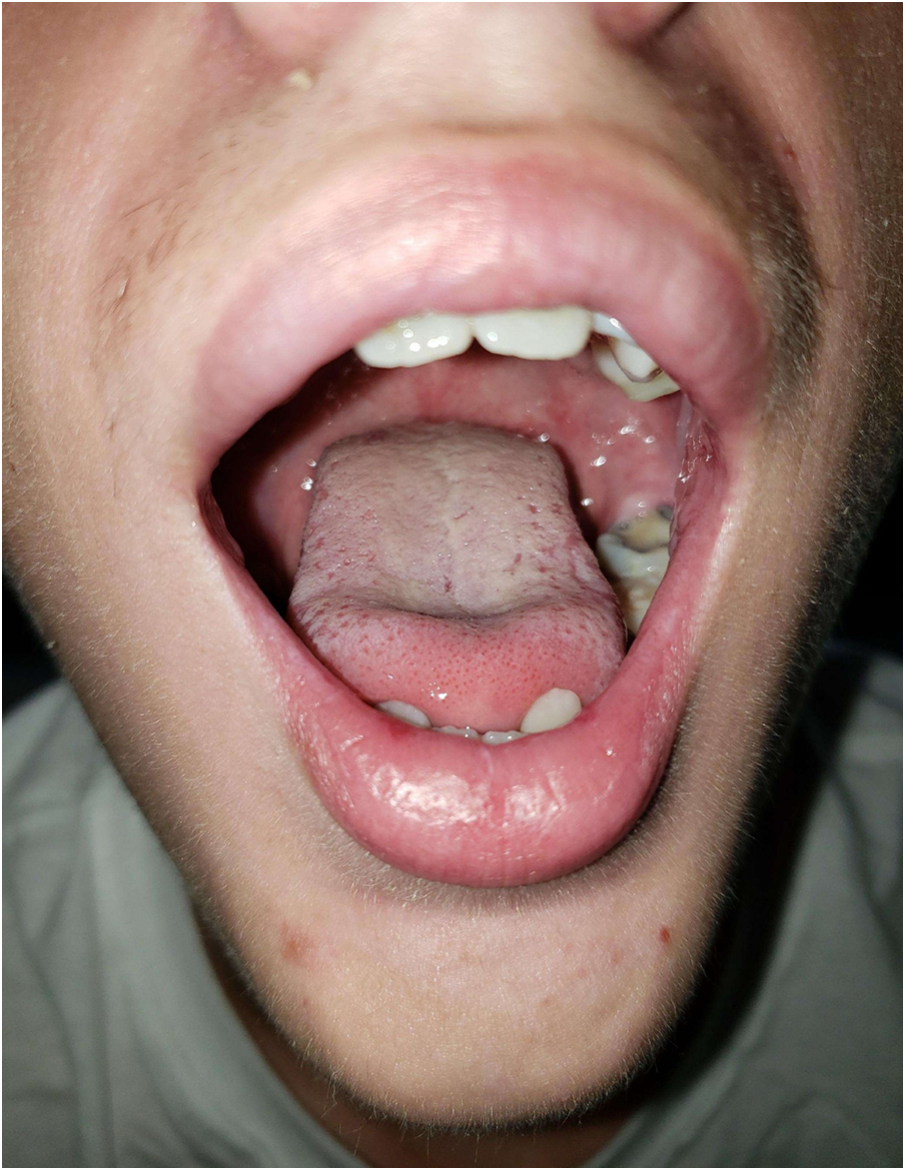
Photographs prior to discharge, 18 days post-diagnosis, extraoral photograph demonstrating healing lesions.

**Figure 4:**
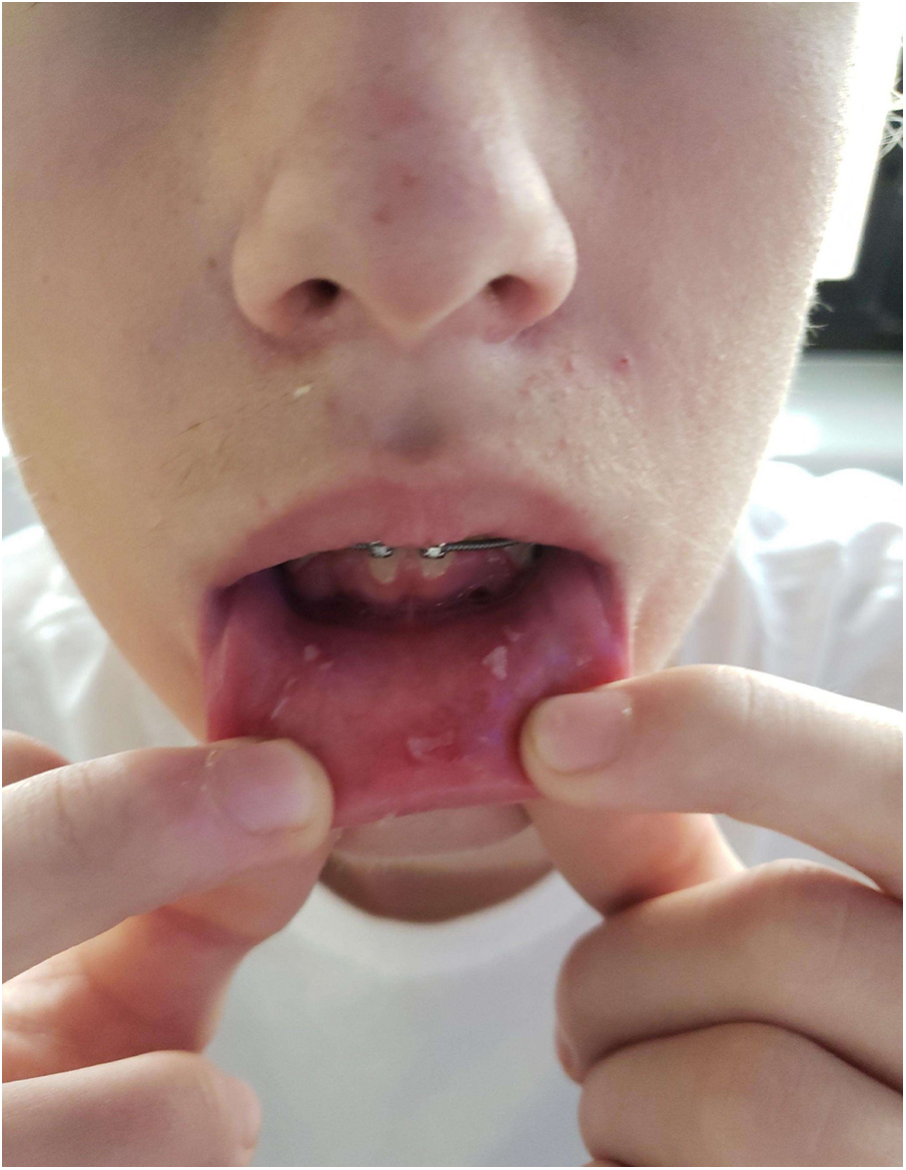
Photographs prior to discharge, 18 days post-diagnosis, intraoral photograph demonstrating healing lesions.

This patient was admitted to a children's hospital for 4 days. He had no improvement with three doses of acyclovir (400 mg tablets) and a topical oral rinse comprised of lidocaine:diphenhydramine:alum-magnesium hydroxide:water in a 1:1:1:2 ratio (one dose tolerated only). He improved after intravenous methylprednisone (60 mg) for 3 days and supportive treatment; pain control with ketorolac, ibuprofen, acetaminophen; fluconazole suspension; and nutritional support including intravenous dextrose and ad lib feeding with Pediasure®, Pedialyte®, and Ensure® (Abbott Laboratories, Abbott, IL, USA). Dental wax was placed over the orthodontic brackets as the patient was experiencing severe pain from the appliance abrading against the oral mucosa. By day 18, the patient had resolution of oral lesions except for remaining ulcers on mucosa of the lower lip (Figures 3, 4).

## 4 Discussion

The differential diagnosis for this patient includes: Stevens-Johnson syndrome (SJS)/toxic epidermolytic necrosis (TEN), erythema multiforme, herpetic gingivostomatitis, or reactive infectious mucocutaneous eruption. Reactive infectious mucocutaneous eruption (RIME) secondary to SARS-CoV-2 infection is consistent with reports in the literature (8–11, 14).

Stevens-Johnson Syndrome/Toxic Epidermal Necrolysis (SJS/TEN) is a continuum of a severe mucocutaneous reaction preceded by fever and caused by an exposure to drugs (15). Lesions were limited to the oral mucosa for the current patient and he did not have lesions present on his trunk which progressed to skin detachment which makes SJS/TEN less likely compared to RIME.

Herpetic gingivostomatitis (HG) is a common clinical manifestation of herpes simplex virus type 1 (HSV-1) (16, 17). The patient in our case presented with similar ulcerative and erosive lesions but they did not appear until after his prodromal symptoms had resolved and most importantly, HSV DNA was not detected in the oral mucosal swab by PCR. Nonetheless, a primary HG infection could not be completely ruled out as a clinical diagnosis due to similarity in symptoms with RIME.

Erythema multiforme (EM) appears to be an immune-mediated entity presenting with an acute onset of classic target-like blistering ulcerative mucocutaneous lesions (18–21). The characteristic target lesions differentiate EM from other conditions and EM differs in cause from SJS/TEN (20). The lack of characteristic target lesion morphology and the clinical appearance inconsistent with oral EM clinically distinguishes EM for our patient compared to RIME. This patient is similar to other cases of RIME associated with COVID-19 in the literature due to the presence of oral lesions in the absence of other mucocutaneous involvement. Some oral findings reported with RIME include erosions, ulcers, vesiculobullous lesions, or denudation of the buccal mucosa (22). This case report is significant in that it involves only the oral mucosa (14, 23). The degree of mucosal involvement in our case report was in the presence of an acute COVID-19 infection without ocular or genital involvement. Given the patient's degree of discomfort and inability to tolerate oral intake, the medical team attempted a trial of IV steroid treatment as initial treatment did not improve symptoms. This is similar in management to other cases of RIME in children, where systemic corticosteroids may benefit the management in combination with pain management, hydration, nutrition, and supportive care (8, 9, 24). However, without an evidence-based guidelines for treatment, a systematic review describes the different approaches in treatment including supportive care, systemic immunosuppression, and/or antibiotics (22, 25). Dental professionals who may encounter similar cases in practice should consider COVID-19 infections in association with oral lesions. Dental professionals can collaborate with the medical team to weigh the risks and benefits of treatment such as systemic immunosuppression, especially if supportive care failed to improve the patient's status.

## 5 Conclusion

Oral lesions associated with COVID-19 infection vary in presentation. This paper adds to the understanding of systemic manifestations of COVID-19 infection to help identify, manage, and coordinate care for patients with these lesions. This paper provides a reference of clinical findings, management, and interdisciplinary collaboration for caring for this patient. The dental team can contribute to the medical care team on appropriate diagnosis, management, and follow-up for patients with COVID-19 infections.

## Data Availability

The original contributions presented in the study are included in the article/Supplementary Material, further inquiries can be directed to the corresponding author.
